# Endotracheal tube cuff pressure assessment maneuver induces drop of expired tidal volume in the postoperative of coronary artery bypass grafting

**DOI:** 10.1186/1749-8090-7-53

**Published:** 2012-06-10

**Authors:** Douglas W Bolzan, Solange Guizilini, Sonia M Faresin, Antonio CC Carvalho, Angelo AV De Paola, Walter J Gomes

**Affiliations:** 1 Department of Medicine, Cardiology Discipline, Pirajussara and São Paulo Hospitals, Escola Paulista de Medicina, Federal University of Sao Paulo, Napoleao de Barros, 715, Sao Paulo, 04024-002, Brazil; 2 Department of Human Movement Sciences, Physiotherapy School, Federal University of Sao Paulo, Ana Costa, 95, Santos, 11060-001, Brazil; 3 Department of Medicine, Pneumology Discipline, São Paulo Hospital, Escola Paulista de Medicina, Federal University of Sao Paulo, Pedro de Toledo, 720, Sao Paulo, 04039-002, Brazil; 4 Department of Medicine, Cardiovascular Surgery Discipline, Pirajussara and São Paulo Hospitals, Escola Paulista de Medicina, Federal University of Sao Paulo, Napoleao de Barros, 715, Sao Paulo, 04024-002, Brazil; 5 Cardiology Discipline, Escola Paulista de Medicina, Federal University of Sao Paulo, Rua Napoleao de Barros, 715, 04024-002, Sao Paulo, Brazil

**Keywords:** Coronary artery bypass grafting, Endotracheal tube cuff pressure, Air leakage, Expired tidal volume, Mechanical ventilation

## Abstract

**Background:**

Previous investigations reported that the cuff pressure (CP) can decrease secondary to the CP evaluation itself. However is not established in literature if this loss of CP is able to generate alterations on expired tidal volume (ETV). Therefore, the aim of this study was to evaluate the potential consequences of the endotracheal CP assessment maneuver on CP levels and ETV in the early postoperative of coronary artery bypass grafting (CABG).

**Methods:**

A total of 488 patients were analyzed. After the operation, the lungs were ventilated in pressure-assist-control mode and the same ventilatory settings were adjusted for all patients. After intensive care unit arrival, the cuff was fully deflated and then progressively inflated by air injection, to promote a minimal volume to occlude the trachea. To assist the cuff inflation and the air leakage identification, the graphical monitoring of the volume-time curve was adopted. After 20 minutes a first cuff pressure evaluation was performed (P1) and a second measurement (P2) was taken after 20 minutes with an analog manometer. ETV was obtained always pre and post P1 measurement.

**Results:**

The CP assessment maneuver promoted a significant drop of P2 in relation to P1 when the manometer was attached to the pilot balloon (p < 0.0001). When compared the moments, pre-P1 versus post-P1, a significant drop of the ETV was also observed (p < 0.0001).

**Conclusion:**

The CP assessment maneuver promoted a significant decrease in CP values and occurrence of air leakage with reduction of ETV in the early postoperative of CABG.

## Background

The main endotracheal tube (ETT) cuff function is to provide an adequate seal of the airway, preventing the air or fluids passage around the ETT. When this seal is compromised, aspirations of pharyngeal contents and poor ventilation may occur, favoring the respiratory complications appearance [1-3].

Previous investigations reported that the cuff pressure (CP) can vary and decrease over the time [4,5]. The maintenance of CP within a therapeutic range is often difficult to obtain, requiring more frequent measurements [6]. However, the frequent measurement can change the CP. The simple maneuver of connecting the pressure monometer to the inflating channel of the pilot balloon can cause a reduction of approximately 2 cmH_2_O in CP [7,8].

Despite the existence of numerous studies involving the importance of assessment and maintenance of the CP within appropriated range, is not yet well established in literature if the loss of CP during measurements is able to generate alterations on expired tidal volume (ETV).

The aim of this prospective study was to evaluate the potential consequences of the ETT CP assessment maneuver on CP levels and ETV in the early postoperative of coronary artery bypass grafting (CABG).

## Methods

This study was conducted at Pirajussara and Sao Paulo Hospitals of the Federal University of Sao Paulo, Sao Paulo, Brazil. After our institutional ethics committee approval and achievement of the written informed consent, a total of 488 patients underwent to elective CABG, from February of 2005 to March of 2010 were prospectively included. Inclusion criteria were age 18 years or older, oral endotracheal intubation, and conventional mechanical ventilation (MV). Patients with obesity, chronic respiratory disease, laryngeal disease or anomaly, and difficult intubation (two or more trials), were excluded from the study.

The lung function indicators of forced vital capacity (FVC) and forced expiratory volume in 1 second (FEV_1_) were evaluated at the bedside on the day before the operation, using a portable spirometer (Spirobank G, MIR, Rome, Italy), according to the standards of the American Thoracic Society to exclude chronic respiratory disease [9].

### Anesthesia and operative technique

Anesthesia was induced in a routine fashion with etomidate and midazolam and maintained with sufentanil and isoflurane. Endotracheal tubes with high residual volume, low-pressure cuff, with an inner diameter of 7.5 mm for female and 8.0 mm for male, were used. The anesthesiologists were free to inflate the ETT cuff as per their clinical judgment and assessment, and blinded to the nature of the study. The patients were ventilated to maintain normocapnia without positive end-expiratory pressure (PEEP) and the inspiratory oxygen fraction (FiO_2_) was maintained between 50% and 60%.

The operation was done on-pump or off-pump, through a midline sternotomy and using the left internal thoracic artery, complemented with additional saphenous vein grafts. In all cases where the pleural cavity was incidentally opened, a pleural drain was inserted. In all patients, a mediastinal tubular drain was also left at the subxyphoid region.

### Postoperative management

After the operation, the patients were transferred to the intensive care unit (ICU), and ventilated in pressure-assist-control mode at 14 breaths/min, inspiratory time of 1.2 seconds, PEEP of 5 cmH_2_O, inspiratory pressure to promote a tidal volume of 8 ml/kg of predicted body weight, and FiO_2_ for keep arterial oxygen saturation above 90%.

### ETT CP interventions

Thirty minutes upon ICU arrival and started MV, a 20 mL syringe was attached to the pilot balloon and the cuff was completely deflated. Sequentially, the cuff was progressively inflated by air injection, to promote a minimal volume to occlude the trachea. To assist the cuff inflation and identify air leakage, the graphical monitoring of the volume-time curve was adopted. The volume-time curve is able to demonstrate the presence of air leak. The existence of an air leak causes decrease of ETV when compared to inspiratory volume. In the presence of air leakage, the descending branch of the volume-time curve does not reach the zero value, with a flattening, being abruptly interrupted by the beginning of the next inspiration [10]. The time of flattening occurrence of the expiratory branch of the curve was used to determine the air leakage. The inflation was performed until the descending branch of the volume-time curve came back to zero, stopping the flattening earlier on. The volume-time curve analysis was performed using a specific device for respiratory mechanics evaluation (Ventcare 9505 VSF, Takaoka, Sao Paulo, Brazil), and utilizing a pressure transducer interposed between the mechanical ventilator circuit and ETT.

Twenty minutes after adjusting of air volume injected into the cuff, ETV was recorded; sequentially the CP was measured and designated as P1. The CP was measured using a manometer graduated in cmH_2_O (VBM Medizintechnik GmbH, Sulz am Neckar, Germany) that was connected to the inflating channel of the pilot balloon. Twenty minutes after P1 measurement, the ETV was monitored. At this point, another measurement of CP, designated P2, was performed to evaluate the actual CP.

After P2 measurement the cuff was re-inflated with air through a 20 mL syringe according to a previously described technique, to avoid any complication resulting from these maneuvers.

During all study period, the patients were maintained in supine position. The head of the bed was kept in elevation of 30 degrees, and the patient's head and neck were maintained in midline with no flexion, extension or rotation.

### Statistical analysis

Data are expressed as means ± standard deviation. The values of ETV post-P1 were expressed as percentage of values obtained pre-P1, as well as the CP values were expressed in percentage (P2 in relation to P1). Data were analyzed using paired Student's *t* test. Statistical analysis was performed with GraphPad Prism 3.0 software (GraphPad Software Inc, San Diego, CA). A value of p < 0.05 was considered statistically significant.

## Results

During the study period, 652 patients were assessed for eligibility. From that sample, 488 were in fact analyzed. Fifteen patients refused to sign the consent form. One hundred forty-nine participants did not meet inclusion criteria. One hundred and four were diagnosed with chronic obstructive pulmonary disease, twenty-two with Obesity, and twenty-three had difficult intubation. Preoperative and intraoperative patients characteristics are summarized in Table 1.

**Table 1:** Preoperative and intraoperative clinical and demographic characteristics

**Variables**	**Patients (n = 488)**
**Age (years)**	63.66 ± 6.84
**BMI**	25.23 ± 1.06
Gender (n)	
Male/female	307/181
Pulmonary function	
FVC (L)	3.47 ± 0.68
% Predicted	96.23 ± 12.41
FEV_1_ (L)	2.72 ± 0.59
% Predicted	95.27 ± 12.53
Operation time (min)	302.14 ± 13.22

BMI = body mass index; FEV_1_ = Forced expiratory volume in 1 second; FVC = forced vital capacity. Data are shown as mean ± standard deviation.

The CP assessment maneuver promoted a significant drop of P2 in relation to P1 when the manometer was attached to the pilot balloon (p < 0.0001) (Table 2). When compared the moments, pre-P1 versus post-P1, a significant drop of the ETV was also observed (p < 0.0001) (Table 3).

**Table 2:** Absolute and percentual values of cuff pressure on P1 and P2

**Variables**	**P1**	**P2**
**Cuff pressure (cmH**_**2**_**O)**	33.14 ± 4.66	30.86 ± 4.60 *
Percentual value (%)		93.00 ± 2.16 **†**

Data expressed as mean ± standard deviation with * p < 0.0001 in relation to P1 versus P2 comparison. P1 = pressure after cuff deflation/re-inflation to appropriated adjustment of pressures, and P2 = pressure after P1 mensuration. **†** Percentage of P2 in relation to P1.

**Table 3:** Absolute and percentual values of expired tidal volume pre-P1 versus post-P1

**Variables**	**Pre-P1 evaluation**	**Post-P1 evaluation**
**Expired tidal volume (mL)**	518.03 ± 52.16	466.95 ± 50.10 *
Percentual value (%)		90.13 ± 3.19 **†**

Data expressed as mean ± standard deviation with * p < 0.0001 in relation to Pre-P1 versus Post-P1 comparison. P1 = pressure after cuff deflation/re-inflation to appropriated adjustment of pressures. **†** Percentage values of post-P1 in relation to pre-P1.

## Discussion

In this prospective study, we evaluated potential consequences of manual CP measurement maneuver in own CP and ETV. The connection of the manometer to the pilot balloon by itself was associated with a significant decrease of CP and reduction of the ETV in this group of patients undergoing CABG on immediate postoperative period.

To our knowledge this is the first study that aimed to evaluate the effects of the CP measurement maneuver on ETV. Previous investigations have reported that the proper cuff inflation ensures delivery of the recommended tidal volume and reduces the risk of subglottic secretions aspiration [1,2,5,11,12]. However, the most of these studies emphasized only the necessity to find a CP to promote the adequate seal of airway. Although two studies [7,8] had reported the consequences of CP measurement maneuver in patients undergoing mechanical ventilation, the main outcome was the drop of CP and not the ETV. Therefore, a novel aspect of our study was to evaluate the impact caused by a single CP assessment maneuver on ETV.

The main function of the ETT cuff is to seal the airway, preventing aspiration of pharyngeal contents into the trachea and leaks around the cuff during positive pressure ventilation [1-3]. In the absence of a precise recommendation, many professionals consider 20 cmH_2_O an acceptable lower limit for CP in adults [5]. Optimal values for cuff pressure are not known, and suggested CP varies from 20 to 30 cmH_2_O [13].

In our study we observed that even after the cuff inflation to promote a minimal volume necessary to occlude the trachea, pressures remained above the level recommended by literature, around 33 cmH_2_O. However this value was enough and therefore needed to stop the air leakage. We believe that this average of pressure found is related to the patients’ tracheal diameter in relation to the ETT size used. The literature have suggested that due to differences in diameter and shape of the human tracheas and also of the ETT size, a higher or lower pressure to seal the airway may be required [14], not necessarily equating to the tracheal wall pressure [15,16]. Carrol and colleagues in experimental study demonstrated that tracheal wall pressure could present lower than intra-CP when high residual and low-pressure cuffs are inflated to seal the airway [15]. In addition, the literature suggests that the limits of the tracheal wall mucosa for blood and lymphatic flow impairment may vary inter- and intra-individuals, depending the systemic arterial pressure and perfusion [17]. In the clinical practice, CP limits are determined in part by the blood pressure of the capillaries that supply the trachea [18]. Elevated CP could result in reduction of the tracheal perfusion, whereas total obstruction of tracheal blood flow occurs with pressures around 50 cmH_2_O [18,19].

McGinnis and co-workers also support our findings. According to these authors the tracheal wall pressure depends in a complex manner of the tracheal size, the relative diameters of the tube and the trachea, and the inflation pressure required to extend the cuff to occlude the trachea. The lowest tracheal wall pressure required is determined by the need to seal the airway against aspiration. They also report that most of the time, tracheal wall pressure excess is unavoidable when extensible occluding cuffs are used on ETT. To avoid complications due to pressure, large residual volume cuffs should be used in combination with a positive means to control and promote a minimal inflation pressure possible [16].

Normally, the CP is measured every 8 to 12 hours, once the CP can decrease naturally over the time, during the attachment of the pressure manometer to the pilot balloon, or due to factors related to patients' own respiratory mechanics [4,20].

There are evidences that CP decreases around 2 cmH_2_O with the connection of a pressure manometer to the pilot balloon [7]. Thus, frequent measurement of CP may increase the risk of complications [7,8]. Similar results are found in our study, the measurement of CP with a manometer device was also related to a significant drop of CP, an average of 2.28 cmH_2_O (7%).

Some authors speculate that the main problem associated with the reduction of CP would be the difficulty to maintain the necessary pressure to ensure adequate lung inflation [20-22]. Therefore, one of the questions of our study was to determine if this drop of CP could influence the decrease of the ETV. We observed that a single CP measurement maneuver was able to generate a significant 9.87% drop on ETV. We believe that this reduction of the ETV is related to the loss of cuff air volume allowing air leak around the cuff.

We speculate that depending of the clinical state, especially in critically ill patients who already present severe hypoxemia, multiple and/or sequential cuff pressure measurements can cause clinical impact. Additional researches are needed to identify the behavior of the cuff pressure, air leakage, exhaled tidal volume and blood gases in relation to sequential evaluation maneuvers and more serious clinical conditions.

## Conclusion

The CP assessment maneuver showed a significant decrease in CP values and air leakage with reduction of ETV when the manometer was attached to the pilot balloon in the early postoperative of CABG.

## Abbreviations

CABG, Coronary artery bypass grafting; CP, Cuff pressure; ETT, Endotracheal tube; ETV, Expired tidal volume; FEV1, Forced expiratory volume in 1 second; FVC, Forced vital capacity; MV, Mechanical ventilation.

## Competing interests

The authors declare that they have no competing interests.

## Authors’ contributions

DWB: Conceived the study and designed the study, analysis and interpretation of data and coordination and wrote the manuscript. SG: Participated in the design of the study, performed the statistical analysis and helped to draft the final manuscript. SMF: Participated in the sequence alignment, have been involved in drafting the manuscript, revising it critically for important intellectual content. ACCC: Participated in the design of the study and performed the statistical analysis. AAVDP: Participated in the design, analysis and interpretation of data. WJG: Conceived the study, coordination and revising it critically for important intellectual content. All authors read and approved the final manuscript.
